# Biological Activities of *Stachys rupestris*, Development of *S. rupestris* Extract-Loaded Alginate Films as Wound Dressing

**DOI:** 10.3390/ph18121868

**Published:** 2025-12-08

**Authors:** Erkan Rayaman, Turgut Taşkın, Elif Çalışkan Salihi, Shalaleh Hasan Niari Niar, Duygu Taşkın, Ceyda Ekentok Atıcı, Ömer Kılıç, Pervin Rayaman, Pelin Özçelik, Hatice Kübra Elçioğlu

**Affiliations:** 1 Department of Pharmaceutical Microbiology, Faculty of Pharmacy, Marmara University, İstanbul 34854, Türkiye; pgocer@marmara.edu.tr; 2 Marmara Pharmacy Drug and Innovative Product Development Unit, Faculty of Pharmacy, Marmara University, İstanbul 34854, Türkiye; turguttaskin@marmara.edu.tr (T.T.); elif.caliskan@marmara.edu.tr (E.Ç.S.); shalaleh.hniariniar@gmail.com (S.H.N.N.); duygu.taskin@sbu.edu.tr (D.T.); ceyda.ekentok@marmara.edu.tr (C.E.A.); kubra.elcioglu@marmara.edu.tr (H.K.E.); 3 Department of Pharmacognosy, Faculty of Pharmacy, Marmara University, İstanbul 34854, Türkiye; 4 Department of Basic Pharmaceutical Sciences, Faculty of Pharmacy, Marmara University, İstanbul 34854, Türkiye; 5 Department of Analytical Chemistry, Faculty of Pharmacy, University of Health Sciences, İstanbul 34668, Türkiye; 6 Department of Pharmaceutical Biotechnology, Faculty of Pharmacy, Marmara University, İstanbul 34854, Türkiye; 7 Department of Pharmaceutical Botany, Faculty of Pharmacy, Adıyaman University, Adıyaman 02040, Türkiye; okilic@adiyaman.edu.tr; 8 Faculty of Pharmacy, Marmara University, İstanbul 34854, Türkiye; peliinozcelik@gmail.com; 9 Department of Pharmacology, Faculty of Pharmacy, Marmara University, İstanbul 34854, Türkiye

**Keywords:** *Stachys rupestris*, antimicrobial effect, antibiofilm effect, alginate film, wound healing

## Abstract

**Background/Objectives**: Regardless of the underlying cause, wound infections are among the most common complications associated with wound formation. The increasing prevalence of antibiotic resistance poses significant challenges in wound management. Due to their favorable therapeutic properties, alginate films have recently emerged as promising biomaterials for wound treatment. **Methods**: The petroleum ether, chloroform, and methanol extracts of the endemic plant *Stachys rupestris* were prepared using the maceration technique. The antimicrobial activity of the extracts and the extract-loaded alginate film was evaluated by agar well diffusion and microdilution assays, while their antibiofilm activity was assessed by crystal violet staining in microplates. The anti-infective potential was investigated using the *Caenorhabditis elegans* infection model, the phytochemical composition was analyzed by HPLC-DAD, and cytotoxicity was determined by the MTT assay. The alginate film was prepared by the solvent casting method and characterized using FTIR spectroscopy and light microscopy. **Results**: All extracts demonstrated antimicrobial activity, with the methanol extract exhibiting the most potent antimicrobial and antibiofilm effects. Quinic acid was identified as the major constituent. Both the methanol extract and the film displayed no cytotoxic effects and showed significant antimicrobial and antibiofilm activities. **Conclusions**: The *S. rupestris* methanol extract-loaded film exhibited strong antimicrobial and antibiofilm properties, indicating its potential as a valuable therapeutic agent in supporting wound healing.

## 1. Introduction

Skin is our largest organ, and it protects our body from various biological, chemical, and physical hazards and excessive loss of moisture. It also plays a vital role in keeping away the harmful microorganisms from the interior of the host. A wound is an injury during which the body wholeness is interrupted by various factors such as infectious agents, acute trauma, or surgery [1,2,3].

It is known that Gram-positive bacteria such as *Staphylococcus aureus*, *Staphylococcus epidermidis*, *Cutibacterium acnes*, and *Pseudomonas aeruginosa* are the primary bacteria found in the skin and can cause various skin disorders, such as skin abscesses, chronic wounds, acne vulgaris, and other inflammatory lesions. Unfortunately, these opportunistic pathogenic bacteria can also cause fatal systemic infections when they enter the bloodstream as they lead to blood poisoning, which can be life threatening for the host. Moreover, these skin microorganisms are able to form various biofilms that are highly persistent and resistant to treatment [4,5,6].

Currently, there are difficulties in the treatment of infections caused by resistant bacteria due to the limited number of antibiotic options that can be used and even the lack of options for some infections. Accordingly, studies on new compounds and methods that can prevent infections and create beneficial effects during treatment are increasing today. In this respect, plants have a high potential in decreasing resistant infections [7].

In addition to antimicrobial activity, microorganisms possess certain virulence factors that play a role in the development of infection. Reduction or elimination of these virulence factors can prevent or suppress the development of infection. One of the most important virulence factors is biofilm, which is a microbial community that is irreversibly embedded in a matrix of extracellular polymeric substance (EPS) produced by microorganisms adhering to each other on living or nonliving surfaces, showing different phenotypes in terms of growth rates and gene transcription. Biofilm-forming bacteria are known to be a thousand times more resistant to antibiotics than planktonic forms. Unfortunately, biofilm can also act as a focal point of infection by creating treatment difficulties [8,9,10,11].

Due to resistance to antimicrobial agents and their side effects, plants can be considered as an option. Hence new compounds, including plants that can stop or slow down the biofilm development of pathogenic microorganisms and may contribute anti-infective effects by treating infectious diseases [12].

The *Stachys* L. genus belongs to the Lamiaceae family, whose members are widespread in almost all parts of the world and are precious in terms of medicine and economy. Due to their rich content of essential oils, *Stachys* species are widely used in Türkiye and around the world in various fields, such as spice, herbal tea, and perfume industries. In addition to the major compounds and essential oil of *Stachys* taxa, germakren-D, caryophyllenes, cadinene, and spatulenol are vital components of this plant [13,14,15]. The genus *Stachys* is known as “mountain tea” in Anatolia and is traditionally used for treatment of skin diseases, ulcers, cancer, respiratory disorders, and kidney diseases due to its antibacterial, anti-inflammatory, antipyretic, antioxidant, and cytotoxic effects [14,15].

*Stachys rupestris* is an endemic species which belongs to the *Stachys* genus. Literature searches reveal that there are not sufficient studies on this plant. Its endemism in Türkiye and its traditional use by the local population highlight the importance of investigating its biological activities and analyzing the compounds responsible for these activities [14,15].

The emergence of resistant infections has led to the search for new antimicrobial agents. The use of medicinal plants in the treatment of many diseases keeps this field up to date. Pharmacological studies have confirmed that extracts or secondary metabolites of *Stachys* taxa have significant antimicrobial effects. *Stachys* taxa have been reported to treat genital tumors, splenic sclerosis, inflammatory tumors, and cancerous ulcers, but there are few studies on their antimicrobial activities [16,17,18,19].

*Caenorhabditis elegans* is a nematode and is often used in genetic studies, but it can also be used as an infection model for microorganisms that are pathogenic to humans. Therefore, *C. elegans* was used as a model organism in our study to determine the anti-infective effect of *S. ruspestris* [20].

Natural film formulations for wound healing applications are made up of natural polymers such as alginate, gelatin, chitosan, and carrageenan, which have high biocompatibility. These natural films have thicknesses varying from nanometers to micrometers, which possess soothing and cooling effects due to their moistening property. Alginate films have attracted interest in recent years owing to their biocompatibility, flexibility, softness, and adaptivity in fastening the wound healing. In addition to these features, alginate films are adhesive, and they could be beneficial during wound healing by transmitting oxygen and carbon dioxide together with water evaporation, which also protects the wound from various microbial infections. Enhancement of the films by antimicrobial agents enriches their antimicrobial activity so that a wound dressing becomes not only effective against bacteria but also against yeast infections. Since it is quite easy to remove the film dressings from the wound, it is favorable to use it during the healing process [3,21,22].

Recently, during the therapy of wounds, there have been some kinds of wound dressing suitable to heal them, few of which are compatible with antimicrobial agents. Unfortunately, the number of resistant microorganisms against these types of dressing material is increasing day by day. Therefore there is a crucial need for new and natural substances such as plant extracts. In our opinion, the collaboration of plant extracts with natural film formulations prepared as a wound dressing could be the solution for the resistance problem stated above [1].

There is no study in the literature related to a formulation consisting of the above-ground parts of *S. rupestris* in wound dressing. Accordingly, we investigated the antimicrobial, antibiofilm, and anti-infective effects of *S. rupestris* methanol extract-loaded film against various microorganisms causing skin disorders.

## 2. Results

### 2.1. Antimicrobial Activity of S. rupestris Extracts and S. rupestris Extract-Loaded Film

In the antimicrobial activity test, the methanol extract obtained from the above-ground parts of *S. rupestris* was used at a concentration of 15 mg/mL. *S. rupestris* petroleum ether (SrPE), methanol (SrM), and chloroform (SrC) extracts exhibited antimicrobial activity especially against Gram-positive microorganisms. Among all of the extracts, the SrM was found to have the strongest antimicrobial activity. The SrM showed antimicrobial effects against *Staphylococcus aureus*, *Staphylococcus epidermidis*, *Cutibacterium acnes*, *Acinetobacter baumannii*, and *Candida albicans* (Table 1). The antimicrobial activity results obtained with *S. rupestris* were lower compared to meropenem and amphotericin B. Additionally, the SrM-loaded film (SrMF) was found to possess antimicrobial activity (Table 2).

**Figure 1:**
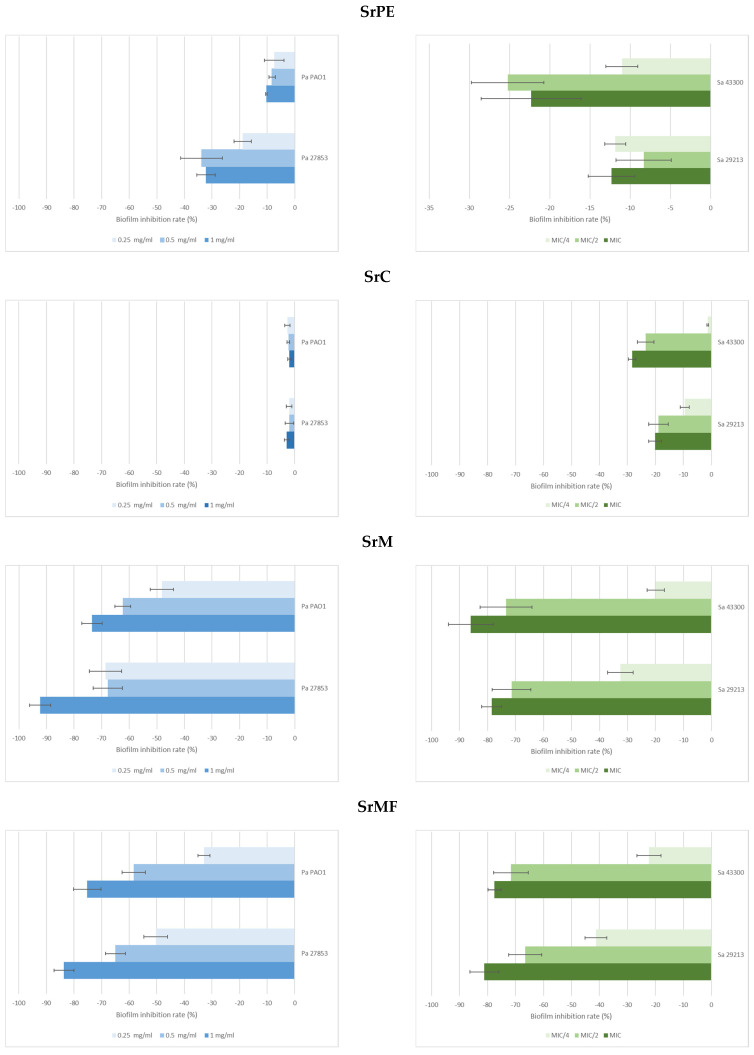
Efficacy of *S. rupestris* petroleum ether extract (SrPE), *S. rupestris* chloroform extract (SrC), *S. rupestris* methanol extract (SrM), and SrM-loaded film (SrMF) on biofilm formation. (Pa 27853: *P. aeruginosa* ATCC 27853, Pa PAO1: *P. aeruginosa* PAO1, Sa 29213: *S. aureus* ATCC 29213, Sa 43300: *S. aureus* ATCC 43300).

The extracts were found to affect biofilm formation of *P. aeruginosa* and *S. aureus* strains in different amounts (Table 3, Figure 1). Although SrPE and SrC had an effect on biofilm formation, this effect was low. The antibiofilm activity of SrM was found to be quite high compared to the other extracts. The activity of SrM on biofilm formation was dose-dependent and strong.

The biofilm inhibition rates of the SrM varied in a dose-dependent manner, ranging from 92.28% to 48.23% for *P. aeruginosa* and from 85.98% to 19.9% for *S. aureus*.

The biofilm inhibition rates of *S. rupestris* extract ranged from 92.28% to 48.23% for *P. aeruginosa* and from 85.98% to 19.9% for *S. aureus* (Table 3). The biofilm inhibition rates of the SrMF varied in a dose-dependent manner, ranging from 83.65% to 32.85% for *P. aeruginosa* and from 81.11% to 22.37% for *S. aureus* (Table 4, Figure 1). SrMF has been found to possess antibiofilm activity on *P. aeruginosa* and *S. aureus* (Table 4, Figure 1).

### 2.2. Detection of Anti-Infective Effect in a C. elegans Model

The *S. rupestris* extracts were found to have no anti-infective activity against infections caused by *P. aeruginosa* and *S. aureus* in the *C. elegans* model (Table 5).

### 2.3. HPLC-DAD Analysis of Phenolic Compounds

**Figure 2:**
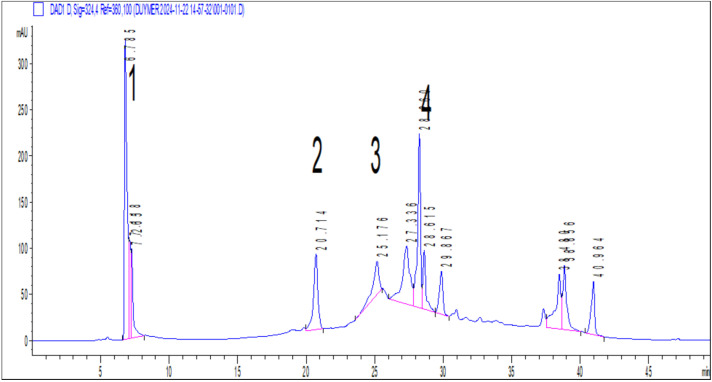
HPLC-DAD chromatogram of SrM at 324 nm. (1: Quinic acid; 2: Chlorogenic acid; 3: 8-OH salvigenin; 4: Rosmarinic acid).

The phenolic composition of *S. rupestris* methanol extract was quantitatively investigated by HPLC. Quinic acid, chlorogenic acid, rosmarinic acid, and 8-OH salvigenin were found in the extract. Among the phenolic compounds, the highest amount of quinic acid (8.65 µg analyte/mg extract) was analyzed in the extract, followed by rosmarinic acid (7.77 µg analyte/mg extract), chlorogenic acid (3.81 µg analyte/mg extract), and 8-OH salvigenin (3.54 µg analyte/mg extract) compounds, respectively (Figure 2 and Table 6).

### 2.4. Cytotoxicity of SrM and SrMF

**Figure 3:**
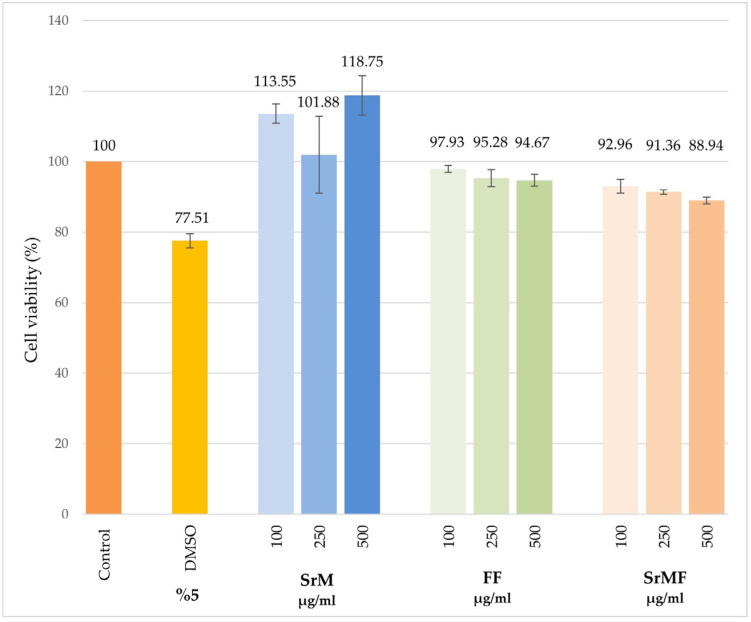
Cytotoxic effect of SrM and SrMF against L929 cells. (SrM: *S. rupestris* methanol extract; FF: Extract-free film; SrMF: SrM-loaded film).

The cytotoxic effect of SrM and SrMF in healthy cells (L929) was determined by MTT assay. According to the test results, both SrM and SrMF at 100–250 and 500 µg/mL final concentrations showed no cytotoxicity on L929 cells (Figure 3).

### 2.5. Characterization of the Alginate Film Formulations

**Figure 4:**
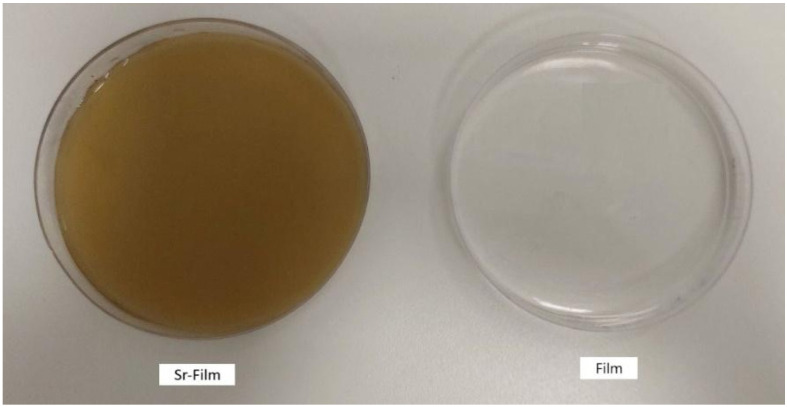
SrMF (Sr-Film) and FF (Film) formulations

**Figure 5:**
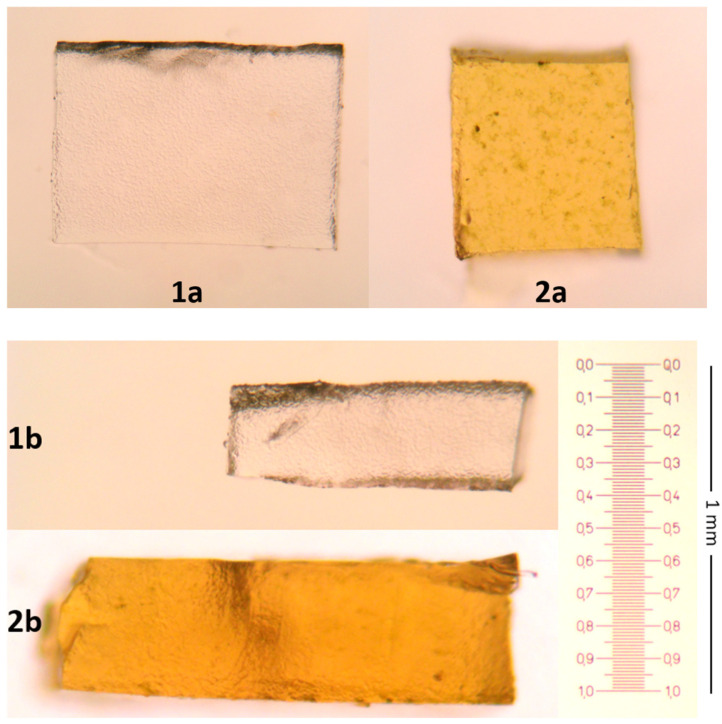
Microscope view of FF and SrMF (40×). Superficial appearance ((**1a**) FF, (**2a**) SrMF), cross-sectional view ((**1b**) FF; (**2b**) SrMF).

The thicknesses of the FF and SrMF (Figure 4) were measured to be approximately 0.3 and 0.4 mm, respectively (Figure 5).

**Figure 6:**
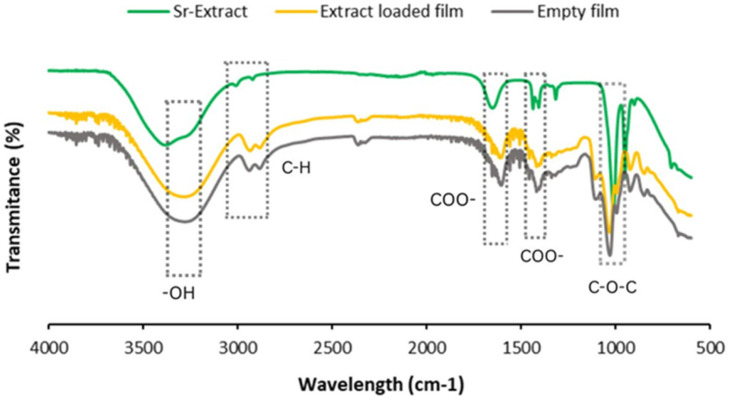
FTIR spectra of SrM (Sr-Extract), SrMF (Extract-loaded film), and FF (Empty film).

The FTIR spectra (Figure 6) show the chemical functionalities of the samples and possible interactions between the active agents in the SrM extract and the film formulation.

**Figure 7:**
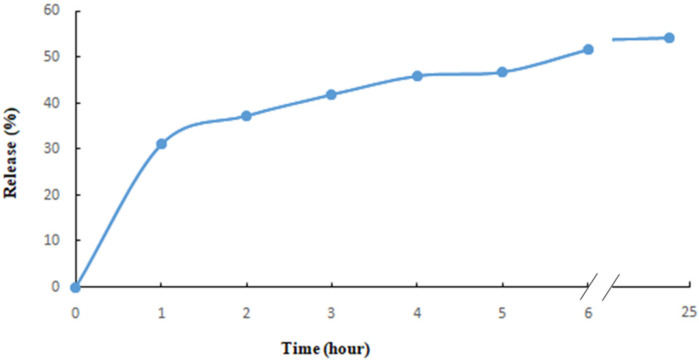
In vitro release profile of SrMF.

In vitro release of SrMF was examined to evaluate the potential use of the produced film formulations in delivery systems of active ingredients, and the release profile is given in Figure 7, which shows the release ability of SrM from the film formulation.

## 3. Discussion

Wounds on the skin may arise from various causes. Contamination of wounds by microorganisms and the subsequent development of infection are common occurrences. Localized infection may develop due to contamination of wounds by microorganisms and various virulence factors of microorganisms, while the spread of pathogens into the bloodstream can cause systemic infections, sepsis, and even death. Moreover, wounds may become chronic, particularly in patients with diabetes and other chronic diseases. Currently, the increasing prevalence of antibiotic resistance further complicates the treatment of infectious diseases. Therefore, regardless of their etiology, wounds require prompt care that provides healing which is non-toxic, prevents infection, and exhibits antimicrobial and antibiofilm properties [6,8,9,12,23,24,25,26,27,28,29,30]. In addition, it is estimated that wound-related problems will increase due to the growing elderly population and the rising prevalence of chronic diseases all over the world [25].

Investigations facilitating the treatment substitutes in wound healing are promptly developing. Improvements in the scope of pharmaceutical and chemical sciences have progressed in the generation of various biomaterials that can rapidly provide tissue reformation and also wound healing. Collaboration of bioactive agents such as plant extracts, vitamins, and peptides, which have antimicrobial properties, with films might fasten the process of wound healing [29,30,31].

Studies on film dressings have demonstrated their beneficial effects on wound healing. When formulated with various plant-derived or other natural substances, these films have been shown to be compatible by enabling the functional activities of such substances to be effectively expressed. For instance, Ehterami et al. [32] demonstrated that D3-loaded alginate hydrogel significantly accelerated the healing of skin wounds in rats. Liakos et al. [33] have reported that essential oils of cinnamon, lavender, tea tree, peppermint, and lemongrass encapsulated in sodium alginate films used as wound dressing possessed antimicrobial properties against *E. coli* and *C. albicans*. Mutlu et al. [34] have stated that *Hypericum perforatum* extract (HPE) incorporated in alginate films (HPE/Al) in various ratios (0.25–1% *v/v*) exhibited antibacterial activity against *E. coli* and *S. aureus*, when compared to the alginate film (Al) alone. Khairan et al. [35] prepared hydrogel films using polyvinyl alcohol, corn starch, patchouli oil, and silver nanoparticles and demonstrated that the films possessed considerable structural stability and were able to retain antimicrobial efficacy. In particular, the formulation containing a light fraction of patchouli oil and silver nanoparticles maintained antimicrobial activity and exhibited strong antimicrobial effects against *S. aureus* and *S. epidermidis*, thereby highlighting their potential as promising antimicrobial biomaterials. Also Candido et al. [36] have stated that alginate hydrogels incorporating neomycin or propolis could be used as wound dressing materials since they act as barriers against microbial penetration due to the hydrogel’s three-dimensional structure.

*Stachys* L. (Lamiaceae) is a huge genus consisting of approximately 300 species spreaded throughout nearly all regions of the world. It is known that the members of this genus have been used as folk medicine for ages in the treatment of cough, ulcers, diarrhea, fever, weakness of the liver and heart, genital tumors, sclerosis of the spleen, and inflammatory diseases. In addition to their role in ethomedicine, some studies confirmed that various extracts of *Stachys* spp. possessed antioxidant, antibacterial, cytotoxic, antitoxic, and anti-inflammatory effects. It is also known that *Stachys* is rich in terms of secondary metabolites, such as flavonoids, iridoids, acetic, lactic, succinic, formic, and malic acids, fatty acids, and phenolic acids [14,37].

*S. rupestris* (Lamiaceae) is an endemic species belonging to the *Stachys* genus [14]. It has been reported that the endemic *S. rupestris* mainly contains α-pinene (22.68%), eucalyptol (4.40%), and terpinen-4-ol (4.36%) [38]. Literature searches reveal that there are not sufficient studies on this plant. Its endemism in Türkiye and its traditional use by the local population highlight the importance of investigating its biological activities and analyzing the compounds contributing to these activities. Therefore in our study we investigated some of the microbiologic effects of *S. rupestris* extract-loaded film.

Limited microbiological studies on *S. rupestris* have generally focused on its essential oil. Ugur et al. [39] reported that the essential oil of *S. rupestris* exhibited antimicrobial activity against *Bacillus subtilis* ATCC 6633, *Bacillus cereus* RSKK 863, *Micrococcus luteus* LA 297, *Stretococcus mutans* CNCTC 8/77, and *S. aureus* ATCC 25923, as well as two multidrug-resistant strains of *S. aureus* and *Stenotrophomonas maltophilia*. Eliuz et al. [15] found that the essential oil of *S. rupestris* exhibited antimicrobial activity against *A. baumannii*, *E. faecalis*, *S. aureus*, *C. albicans*, and *C. tropicalis*, while showing no antimicrobial effect against *E. coli*. Furthermore, the researchers contaminated lab-made skin with these microorganisms to investigate the effect of *S. rupestris* essential oil on microbial counts. Treatment with the essential oil reduced the number of all tested microorganisms by 59.3–92.2%, thereby demonstrating its potential application in reducing microbial contamination on skin surfaces. As mentioned above, it has been determined that *S. rupestris* essential oil has antimicrobial properties and causes serious reductions in the number of some microorganisms contaminated on artificial skin [15].

In our study, the *S. rupestris* extracts showed antimicrobial activity, especially against *S. aureus*, *S. epidermidis*, and *C. albicans*. In addition, the SrM also showed antimicrobial activity against *C. acnes* and *A. baumannii* (Table 1). It is known that Staphylococcal species are commonly found on the skin and are one of the main agents of skin infections [28]. The activity of SrM on *S. aureus* suggests that it may be useful for healing wound infections. In addition, the antimicrobial effect of *S. rupestris* methanol extract on *C. acnes* showed that it has the potential to be used in acne treatment. It was found that the film formulation obtained maintained the antimicrobial activity of *S. rupestris* extract and showed activity close to the extract (Table 2).

In the anti-infectivity study conducted using the *C. elegans* model, no activity was detected in any of the *S. rupestris* extracts (Table 5). We believe that this may be due to the limitations of the model.

In the determination of the antibiofilm activity of *S. rupestris* extracts on *P. aeruginosa*, 1–0.25 mg/mL concentrations of the extracts were used since no antimicrobial activity was detected (Table 3, Figure 1). In general, although inhibition was detected in all *S. rupestris* extracts, antimicrobial activity was limited in petroleum ether and chloroform extracts. In terms of antibiofilm activity, the SrM showed a very strong activity (Table 3, Figure 1). Although no antimicrobial activity was detected against *P. aeruginosa*, the highest antibiofilm activity was 92.28% against *P. aeruginosa* and 85.98% against *S. aureus*. Although SrMF is less effective than SrM, it has been observed that SrM possesses antibacterial properties (Table 3 and Table 4, Figure 1).

It was determined that SrM and SrMF did not have toxic properties (Figure 3). This statement indicates that the film does not have a negative effect on wound healing.

In the present study the contents of the methanol extract by HPLC-DAD were found to be quinic acid, rosmarinic acid, 8-OH salvigenin, and chlorogenic acid, respectively. Quinic acid in the extract composition has previously been reported to have antimicrobial activity against *S. aureus* and *E. coli* and antibiofilm activity against *P. aeruginosa*, and to increase the effectiveness of kanamycin against *S. aureus* [40,41,42]. Rosmarinic acid and its derivatives are known to have strong antimicrobial activity against many pathogens. In our study, it was found that they showed antimicrobial activity against *S. aureus*, *S. epidermidis*, *P. aeruginosa*, *E. coli*, *S. typhimurium*, *K. pneumoniae*, *E. faecalis*, and *C. albicans*. It has also been reported that rosmarinic acid showed antibiofilm activity [43,44]. In a study on 8-hydroxy-salvigenin, no activity was found against *S. aureus* and *S. epidermidis*, while it was found to have antimicrobial activity against *E. coli*, *Proteus vulgaris*, *P. aeruginosa*, *C. albicans*, *Candida glabrata*, *Candida guilliermondii*, *Candida parapsilosis*, and *Candida krusei* [45]. It was shown that chlorogenic acid had antimicrobial activity against many microorganisms, such as *S. aureus*, *P. aeruginosa*, *E. coli*, *K. pneumoniae*, *A. baumannii*, and *C. albicans* and has also been found to exhibit antibiofilm activity against *S. aureus*, *P. aeruginosa*, and *C. albicans* [46,47,48,49]. The activity of the components in the methanol extract of *S. rupestris* explains the effectiveness of the findings we obtained.

FTIR spectra were recorded to understand the chemical functionalities of the produced film formulations, in addition to possible interactions between the phytochemical content of the SrM and alginate structure of the film formulation (Figure 6). A wide absorption band at around 3300 cm^−1^, corresponding to the stretching of OH groups, is seen in the spectra of both films showing the OH groups of the alginate structure; C-H vibration bands are seen at around 2900 cm^−1^; asymmetric and symmetric stretching vibrations of the COO^−^ groups are seen at around 1620 cm^−1^ and 1420 cm^−1^; C-O-C stretching is also seen at around 1040 in Figure 6, which is characteristic of the alginate structure. The FTIR spectra of the Sr extract also has a wide absorption band at around 3400 cm^−1^ which shows the OH groups and is characteristic for polyphenolic compounds. C-H vibrations are seen at around 2900 cm^−1^ with slight peaks. Asymmetric and symmetric vibrations of the COO^−^ groups are available at around 1650 cm^−1^ and 1450 cm^−1^ while C-O-C bonds are seen at around 1000 cm^−1^ [50,51,52,53,54,55,56]. Figure 6 also shows that there are no significant changes in the peak positions when one compares the spectra of SrMF and FF, taking into account the FTIR spectra of Sr, which shows the absence of chemical interaction between the content of SrM and the alginate structure and therefore the presence of a physical dispersion of the extract through the structure. In vitro release of SrMF was examined to evaluate the potential use of the produced film formulations in the delivery systems of active ingredients, and the release profile is given in Figure 7, which shows the release ability of SrM from the film formulation. It is seen from Figure 7 that there is a gradual and slow release of the active ingredient of the formulation where more than 50% of the extract was released during the first six hours. Slow and sustainable drug release of a potential wound dressing is important to improve wound healing through the increase in the duration of the release of the active ingredients. Long release time also improves the compliance of the therapeutic application to the patients since it would increase the application time before replacement [54,55].

## 4. Materials and Methods

### 4.1. Procurement of the Plant Material

**Figure 8:**
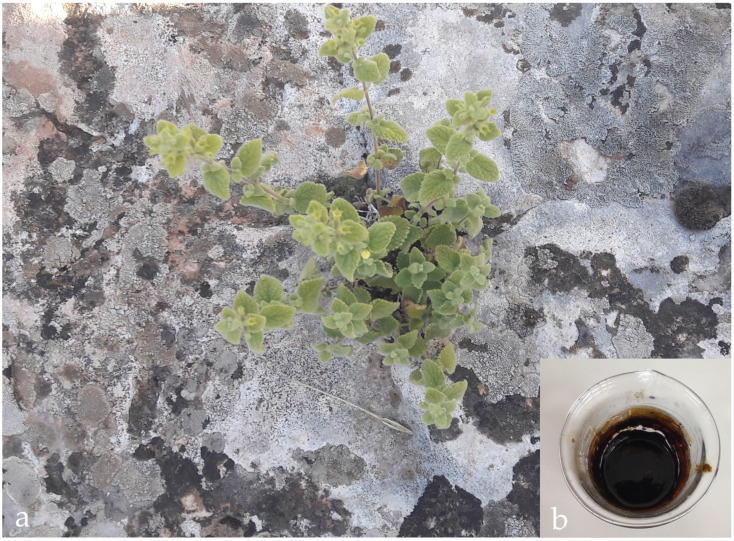
*S. rupestris* (**a**) and its methanol extract (**b**).

*S. rupestris* was collected from the surrounding district of Arsuz (Hatay-Türkiye), from coniferous forest in intervals, during June 2022, and identified by plant taxonomist Dr. Ömer Kılıç (ÖK 1215). Plant samples are deposited in Herbarium of Adıyaman University Faculty of Pharmacy and Yıldırımlı Herbarium in Ankara (Türkiye) (Figure 8a).

### 4.2. Preparation of S. rupestris Extracts

The above-ground parts of *S. rupestris* were dried at room temperature and ground into powder. The above-ground parts were treated sequentially with petroleum ether, chloroform, and methanol by the maceration method, and extracts were prepared. After the maceration process, the liquid portion was filtered through filter paper and collected. The solvents of the filtrates were evaporated in a rotary evaporator (Heidolph Hei-VAP Expert, Schwabach, Germany)and then lyophilized (Faithful FSF-10N-50C, Ningbo, China) to obtain crude extracts. The extracts were stored at 4 °C until the day the study began (Figure 8b) [57,58]. The extract was dissolved in DMSO and used in the experiments.

### 4.3. Antimicrobial Activity

The antimicrobial activity of the plant extracts was first determined by the agar well diffusion method. Minimal inhibitory concentration (MIC) was determined for the extracts showing antimicrobial activity in the agar well diffusion method.

#### 4.3.1. Microorganisms

In our study, microorganisms that cause general and skin infectious diseases were preferred. *Staphylococcus aureus* ATCC 43300, *Staphylococcus aureus* ATCC 29213, *Staphylococcus epidermidis* ATCC 12228, *Streptococcus mutans* ATCC 25175, *Enterococcus faecalis* ATCC 29212, *Cutibacterium acnes* ATCC 11827, *Pseudomonas aeruginosa* ATCC 27853, *Pseudomonas aeruginosa* PAO1, *Klebsiella pneumoniae* ATCC 4352, *Proteus vulgaris* ATCC 13315, *Salmonella typhimurium* ATCC 25175, *Acinetobacter baumannii* ATCC 19606, and *Escherchia coli* ATCC 25922 were the bacterial strains and *Candida albicans* ATCC 90028 was the yeast strain used in this study.

#### 4.3.2. Agar Well Diffusion Method

*S. mutans* ATCC 25175 was grown on Brain Heart Infusion agar at 37 °C for 24 h; *C. acnes* ATCC 11827 was grown on tryptic soy agar containing 5% blood for 48 h at 37 °C under anaerobic conditions; *C. albicans* ATCC 90028 was grown on Sabouraud dextrose agar (SDA) at 37 °C for 48 h; and other microorganisms were grown on tryptic soy agar at 37 °C for 24 h (Memmert INE 500, Büchenbach, Germany). Microorganism suspensions were prepared from the colonies on solid media in 0.85% physiological saline solution (PSS). Bacterial suspensions were adjusted to a concentration of 10^8^ CFU/mL and yeast suspension to a concentration of 10^6^ CFU/mL according to McFarland 0.5 standard turbidity. An aliquot of 100 μL was taken from these suspensions and spread on the surface of Mueller Hinton Agar (MHA) for bacteria and SDA for yeast by sterile swab. Wells with a diameter of 5 mm were made on the medium using a sterile punch at certain intervals, and 50 μL of the extracts (15 mg/mL) dissolved in DMSO was placed into the wells. In addition, meropenem (10 μg/well) for bacteria and amphotericin B (100 μg/well) for yeasts, as well as the DMSO and physiological saline, were used as controls. Petri dishes were incubated at 37 °C for 18–24 h for bacterial growth and at 35 °C for 24–48 h for yeast growth, and growth inhibition zones were measured in mm. The experiments were performed in triplicate and averaged [59,60]. To demonstrate the antimicrobial activity of the film, the film was dissolved in DMSO, and 200 µL sample volumes were used in the agar well diffusion method.

#### 4.3.3. Minimal Inhibitory Concentration (MIC) Determination for Bacteria

A bacterial suspension was prepared in accordance with the Clinical and Laboratory Standards Institute (CLSI) standards. Here the bacterial culture was prepared from colonies according to McFarland 0.5 turbidity and diluted to a final inoculum concentration of 5 × 10^5^ CFU/mL. In sterile U-bottom microdilution plates, 100 μL of Mueller Hinton Broth (MHB) was added. The solubilized extracts were placed in 100 μL amounts into the first wells, respectively, and serial dilutions were made. Then, 5 μL of bacterial suspension was added to the wells containing extracts and incubated at 37 °C for 24 h. At the end of incubation, the lowest sample concentrations without growth were determined as minimal inhibitory concentration (MIC). MHB, DMSO, and meropenem were used as controls [61]. To determine the MBC, 10 μL was taken from the wells showing no visible microbial growth and inoculated onto TSA, followed by incubation. After incubation, the lowest concentration at which no bacterial growth was observed was recorded as the MBC.

#### 4.3.4. Minimal Inhibitory Concentration (MIC) Determination for Yeast

Dilutions of the tested extracts were prepared in U-based microdilution plates in RPMI-1640 medium. *C. albicans* ATCC 90028 was seeded on SDA medium and incubated at 37 °C for 48 h. Then, suspensions were prepared from the cultures according to McFarland 0.5 (1 × 10^6^–5 × 10^6^ yeast/mL) turbidity in RPMI-1640, diluted with RPMI-1640 to 5 × 10^2^–2.5 × 10^3^ yeast/mL, and 100 μL suspension was added to the wells containing extracts. The prepared plates were incubated at 37 °C for 24–48 h. After 24 h of incubation, the plates were evaluated and incubated again and evaluated again at 48 h. The lowest concentrations without growth were considered as minimal inhibitory concentrations. RPMI-1640, DMSO, and amphotericin B were used as controls [62]. To determine the MFC, 10 μL was taken from the wells showing no visible microbial growth and inoculated onto SDA, followed by incubation. After incubation, the lowest concentration at which no yeast growth was observed was recorded as the MFC.

### 4.4. Effect of Samples on Biofilm Formation

*P. aeruginosa* ATCC 27853, *P. aeruginosa* PAO1, *S. aureus* ATCC 43300, and *S. aureus* ATCC 29213 were inoculated in 5 mL of tryptic soy broth-1% glucose (TSBG) medium and incubated at 37 °C for 24 h. Bacterial suspensions equivalent to McFarland 0.5 turbidity standard were prepared from the cultures in TSBG. The prepared suspensions were diluted with 1% TSB-glucose medium to a final concentration of 5 × 10^5^ CFU/mL. The prepared bacterial suspensions were distributed as 180 μL into the wells of the flat bottom microplate. According to the MIC values of the samples against each strain tested, the final concentrations of the samples in the wells were prepared in TSBG at 1, 1/2, and 1/4 times the MIC value. Each of the prepared samples was added as 20 μL to the respective wells containing bacteria in the plate. The prepared plates were incubated at 37 °C for 24 h, and after incubation, the wells were carefully emptied with an automatic micropipette and washed twice with 250 μL phosphate buffer solution (PBS). After washing, 200 μL of 99% methanol was added to the wells and left for 15 min; the wells were emptied, and the microplate was left to dry. After the microplate was dried, 0,1% crystal violet (200 μL) was added to the wells and kept for 5 min, the excess dye was washed away with water, and the microplate was allowed to dry. Then, 200 μL of 95% ethanol was added to the wells and left for 30 min, and at the end of the time, optical density values were measured by the microplate reader (Biotek Epoch, Winooski, VT, USA) at 595 nm in the microplate reader. The experiments were conducted in triplicate, and the arithmetic means of the optical density (OD) values of each strain incubated with the samples at different concentrations were taken. DMSO and extract-free film were used as a control. According to the method applied, the optical density values of the negative and positive control wells were measured, and the mean value was taken. The positive control OD value of each strain was accepted as 100%, and the % value corresponding to the OD values of the wells containing different concentrations of sample was determined according to this value. The absorbance values of the wells containing *S. rupestris* extract-loaded film (SrMF) were subtracted from the absorbance value of the negative control. The change in biofilm formation rate of strains in the presence of different concentrations of *S. rupestris* extract-loaded film was calculated by the following formula [63,64].

Biofilm change rate = (OD_E_/OD_C_) × 100

OD_E_ = OD value of wells containing different concentrations of samples.

OD_C_ = OD value of the positive control well.

### 4.5. Detection of Anti-Infective Effect in C. elegans Model

*C. elegans* AU37 was used as a model organism, and *P. aeruginosa* ATCC 27853 and *S. aureus* ATCC 29213 were used as infective agents to determine the anti-infective effect. Briefly, *C. elegans* was grown on nematode growth medium (NGM) containing *Escherichia coli* OP50 at 16 °C. *C. elegans* was then synchronized in order to detect the anti-infective effect. NGM medium containing SrPE, SrC, and SrM was prepared at a MIC/2 concentration for *S. aureus* ATCC 29213 and at a 1 mg/mL concentration for *P. aeruginosa* ATCC 27853 and then distributed into 24-well microplates. *E. coli* OP50 was added to the wells. On average, 20–30 pieces of synchronized *C. elegans* were distributed in the wells of the microplate, and *P. aeruginosa* ATCC 27853 and *S. aureus* ATCC 29213 were added separately. The microplates were incubated at 25 °C, and the live count of *C. elegans* was performed daily by using a stereo microscope. The counting process was continued until all *C. elegans* died. Extract-free medium, DMSO, and *E. coli* OP50 were used as controls. The comparison of the groups’ lifespans was performed using Kaplan–Meier survival analysis with GraphPad 6.0 software [65,66,67,68].

### 4.6. HPLC Analysis of Phenolic Compounds

HPLC-DAD was used to analyze the phenolic compounds that were present in the extract or extracts and exhibited high biological activity. The system’s operating conditions are listed below. Conditions for HPLC operation: Waters Novapak C18 column, 4 μm (3.9 × 150 mm), water, and 0.05% formic acid made up the mobile phase (A); acetonitrile and 0.05% formic acid made up mobile phase (B). The following gradient program was used: 5% B for 0 min, 5% B for 1 min, 30% B for 20 min, 60% B for 25 min, 60% B for 28 min, 95% B for 33 min, 95% B for 35 min, and 5% B for 40 min. The system was injected with 20 μL of the sample at a flow rate of 0.5 mL/min. After passing through a 0.45 μm syringe tip microfilter, the extracts were diluted in methanol solvent and then added to the HPLC apparatus (Agilent 1260 infinity I, Santa Clara, CA, USA) [69].

### 4.7. Production of the Film Formulations

SrMF and FF were produced by using the solvent casting method [70] modified in our laboratory. The amounts of 300 mg Sr and 300 mg sodium alginate were weighed and dissolved in 10 mL DI water, separately. Two aqueous solutions were mixed using a magnetic stirrer, and 1 mL of glycerin was added to this mixture as a plasticizer to increase the flexibility of the films. Then, the obtained mixture was cast into the Petri dishes followed by drying at 40 °C in an oven. Empty film (FF) was also produced using the same procedure.

### 4.8. Characterization of the Film Formulations

The thickness, surface, and cross-sectional images of the films were determined by light microscopy (Olympus BX50F-3, Kyoto, Japan). Fourier transform infrared (FTIR) spectra were recorded using an IRSpirit spectrometer, Shimadzu Corp, Kyoto, Japan between 4000 and 500 cm^−1^ with an average resolution of 4 cm^−1^. In vitro release of the films was studied spectrophotometrically (Shimadzu 2100S, Kyoto, Japan) by using dialysis bags in PBS (phosphate-buffered saline, pH 7.4) medium. For the release experiment, 25 mg of each film and 50 mL of PBS were used. The release experiment was conducted in a thermostatic shaking water bath at 37 °C. Samples were taken at predetermined time intervals (at 1 h, 2 h, 3 h, 4 h, 5 h, and 6 h), and the concentrations of the samples were calculated using the spectrophotometric method by using the calibration curves prepared initially [71].

### 4.9. Cytotoxicity of SrM, FF, and SrMF

The cytotoxic effect of the extracts and films was determined by using the 3-(4,5-Dimethylthiazol-2-yl)-2,5-Diphenyltetrazolium Bromide (MTT) assay technique. *S. rupestris* methanol extract and film samples were prepared in DMSO, and their effects on cell viability were investigated using the Cell Proliferation Kit I (MTT kit) (Roche, Basel, Switzerland) in the L-929 (ATCC CCl-1) cell line, according to the manufacturer’s instructions. For this purpose, L929 cells were cultured in DMEM supplemented with 10% FBS and then seeded in a 96-well plate at a density of 5 × 10^3^ cells/well and cultured overnight at 37 °C with 5% CO_2_. The extract, FF, and SrMF samples were applied to each well at 100–250–500 µg/mL final concentrations and incubated 24 h. After that, the culture medium was replaced with fresh medium, and 10 µL MTT solution was added to each well. At the end of the 4 h incubation, formazan crystals were solubilized by adding 100 µL of solubilization buffer, the absorbance was measured spectrophotometrically at 550 and 690 nm, and cell viability was calculated in percentage compared to the untreated control group. All samples were studied in quadruplicate.

## 5. Conclusions

In the present study, the methanol extract prepared from the aerial parts of *S. rupestris* (SrM) was found to possess strong antimicrobial and antibiofilm properties. The extract was mainly composed of quinic acid, rosmarinic acid, and 8-OH salvigenin. Based on these findings, an alginate film was prepared using the SrM. The resulting film exhibited antimicrobial and antibiofilm activities and showed no cytotoxicity. The results of our study indicate that the newly developed SrM-loaded film is a promising candidate for use as an antibacterial, antibiofilm, and non-toxic wound dressing for various types of wounds.

## Figures and Tables

**Table 1:** Antimicrobial activity of *S. rupestris* extracts on various microorganisms.

	Agar Well Diffusion Test					Microdilution Test			
	ZD (mm)					MIC (mg/mL)/MBC-MFC * (mg/mL)			
	SrPE	SrC	SrM	DMSO (15%)	M/A	SrPE	SrC	SrM	DMSO (%)
*Staphylococcus aureus* ATCC 43300	14.08 ± 0.31	11.36 ± 0.26	14.52 ± 0.33	0	34.18 ± 0.28/-	0.03/0.12	0.12/0.94	0.03/0.94	>7.5/>7.5
*Staphylococcus aureus* ATCC 29213	12.46 ± 0.22	12.30 ± 0.12	12.76 ± 0.28	0	37.42 ± 0.32/-	0.12/7.5	0.12/7.5	0.12/3.75	>7.5/>7.5
*Staphylococcus epidermidis* ATCC 12228	18.16 ± 0.09	13.99 ± 0.22	20.34 ± 0.32	0	55.44 ± 0.13/-	0.12/3.75	0.47/1.88	0.12/0.94	>7.5/>7.5
*Streptococcus mutans* ATCC 25175	0	0	0	0	52.54 ± 0.19/-	-	-	-	>7.5/>7.5
*Enterococcus faecalis* ATCC 29212	0	0	0	0	22.52 ± 0.10/-	-	-	-	>7.5/>7.5
*Cutibacterium acnes* ATCC 11827	0	0	11.72 ± 0.16	0	64.78 ± 0.32/-	-	-	0.12/0.47	>7.5/>7.5
*Pseudomonas aeruginosa* ATCC 27853	0	0	0	0	32.22 ± 0.11/-	-	-	-	>7.5%/>7.5
*Pseudomonas aeruginosa* PAO1	0	0	0	0	30.29 ± 0.31/-	-	-	-	>7.5/>7.5
*Klebsiella pneumoniae* ATCC 4352	0	0	0	0	36.23 ± 0.09/-	-	-	-	>7.5/>7.5
*Proteus vulgaris* ATCC 13315	0	0	0	0	41.28 ± 0.21/-	-	-	-	>7.5/>7.5
*Salmonella typhimurium* ATCC 25175	0	0	0	0	30.36 ± 0.33/-	-	-	-	>7.5/>7.5
*Acinetobacter baumannii* ATCC 19606	0	0	9.61 ± 0.29	0	32.65 ± 0.35/-	-	-	0.94/1.88	>7.5/>7.5
*Escherchia coli* ATCC 25922	0	0	0	0	32.34 ± 0.17/-	-	-	-	>7.5/>7.5
*Candida albicans* ATCC 90028	12.47 ± 0.21	11.64 ± 0.11	8.20 ± 0.22	0	-/23.98 ± 0.30	0.47/0.94 *	0.47/0.94 *	1.88/3.75 *	>3.75/>3.75

ZD: Inhibition zone diameter, -: Not done, SrPE: *S. rupestris* petroleum ether extract, SrM: *S. rupestris* methanol extract, SrC: *S. rupestris* chloroform extract, M: Meropenem, A: Amphotericin B, -: Not done, MIC: Minimal inhibitory concentration, MBC: Minimal bactericidal concentration, MFC: Minimal fungicidal concentration, *: MFC.

**Table 2:** Antimicrobial activity of SrM and SrM-loaded film (SrMF) using the agar well diffusion method.

	ZD (mm)			
	SrM	SrMF	FF	DMSO (15%)
*S. aureus* ATCC 43300	19.27 ± 0.19	15.70 ± 0.16	0	0
*S. aureus* ATCC 29213	19.01 ± 0.24	14.65 ± 0.20	0	0
*S. epidermidis* ATCC 12228	22.65 ± 0.23	21.78 ± 0.11	0	0
*C. acnes* ATCC 11827	17.42 ± 0.19	16.38 ± 0.22	0	0
*A. baumannii* ATCC 19606	25.36 ± 0.12	24.56 ± 0.23	0	0
*C. albicans* ATCC 90028	20.76 ± 0.18	19.87 ± 0.10	0	0

ZD: Inhibition zone diameter, SrM: *S. rupestris* methanol extract, SrMF: SrM-loaded film, FF: Extract-free film.

**Table 3:** Effect of *S. rupestris* extracts on biofilm formation of microorganisms.

Biofilm Inhibitory Rate (%)												
	SrPE			SrC			SrM			DMSO		
**Concentration (mg/mL)**	**1** *	**0.5**	**0.25**	**1** *	**0.5**	**0.25**	**1** *	**0.5**	**0.25**	**1%**	**0.5%**	**0.25%**
*P. aeruginosa* ATCC 27853	32.18 ± 3.34	33.88 ± 7.52	18.84 ± 3.12	2.92 ± 0.89	1.95 ± 1.58	2.01 ± 1.04	92.28 ± 3.80	67.76 ± 5.29	68.60 ± 5.90	2.32 ± 1.45	−1.66 ± 1.33	−2.12 ± 2.02
*P. aeruginosa* PAO1	10.3 ± 0.22	8.22 ± 1.18	7.45 ± 3.55	1.93 ± 0.61	2.31 ± 0.45	2.66 ± 0.96	73.56 ± 3.69	62.32 ± 2.85	48.23 ± 4.23	1.67 ± 1.86	2.01 ± 0.95	−2.01 ± 1.12
**Concentration** **(mg/mL)**	**0.12 (MIC)**	**0.06 (MIC/2)**	**0.03 (MIC/4)**	**0.12 (MIC)**	**0.06 (MIC/2)**	**0.03 (MIC/4)**	**0.12 (MIC)**	**0.06 (MIC/2)**	**0.03 (MIC/4)**	**0** **.24%**	**0.12%**	**0.06%**
*S. aureus* ATCC 29213	12.3 ± 2.89	8.32 ± 3.45	11.85 ± 1.32	20.1 ± 2.32	18.9 ± 3.45	9.5 ± 1.65	78.56 ± 3.58	71.45 ± 6.89	32.56 ± 4.56	3.41 ± 2.01	2.56 ± 1.22	3.13 ± 0.93
**Concentration** **(mg/mL)**	**0.03 (MIC)**	**0.015 (MIC/2)**	**0.008 (MIC/4)**	**0.12 (MIC)**	**0.06 (MIC/2)**	**0.03 (MIC/4)**	**0.03 (MIC)**	**0.015 (MIC/2)**	**0.008 (MIC/4)**	**0.24%**	**0.12%**	**0.06%**
*S. aureus* ATCC 43300	22.32 ± 6.23	25.23 ± 4.52	11.02 ± 1.98	28.36 ± 1.36	23.5 ± 2.98	1.3 ± 0.23	85.98 ± 7.98	73.36 ± 9.28	19.9 ± 3.12	1.91 ± 1.23	−2.01 ± 1.09	2.61 ± 2.13

SrPE: *S. rupestris* petroleum ether extract, SrC: *S. rupestris* chloroform extract, SRM: *S. rupestris* methanol extract, MIC: Minimal inhibitory concentration, * This concentration was used because no antimicrobial activity was detected.

**Table 4:** Effect of SrMF on biofilm formation of microorganisms.

	Biofilm Inhibitory Rate (%)			
	SrMF			DMSO (1%)
*P. aeruginosa* ATCC 27853	83.65 ± 4.12	65.01 ± 3.62	50.38 ± 4.23	2.01 ± 1.35
*P. aeruginosa* PAO1	75.18 ± 5.01	58.39 ± 4.19	32.85 ± 2.19	2.31 ± 2.11
*S. aureus* ATCC 29213	81.11 ± 5.21	66.54 ± 5.88	41.28 ± 3.88	−2.54 ± 1.45
*S. aureus* ATCC 43300	77.49 ± 2.37	71.65 ± 6.18	22.37 ± 4.31	−1.76 ± 1.21

**Table 5:** Anti-infective effect of *S. rupestris* extracts in *C. elegans* model.

	Life Span of *C. elegans* (Day)				
	Control	SrPE	SrC	SrM	DMSO (1%/0.24%)
*P. aeruginosa* ATCC 27853	22.33 ± 1.53	21.33 ± 1.53 *	24.33 ± 2.51 *	19.66 ± 2.08 *	23.12 ± 1.12 */22.28 ± 1.10 *
*S. aureus* ATCC 29213	23.33 ± 2.08	23.33 ± 2.08 *	21.66 ± 1.51 *	25.66 ± 1.84 *	22.18 ± 1.32 */23.42 ± 1.34 *

* *p* > 0.05, SrPE: Petroleum ether extract, SrC: Chloroform extract, SrM: Methanol extract.

**Table 6:** Chemical compounds of SrM.

Compounds	µg Analyte/mg Extract
Quinic acid	8.65 ± 1.18
Rosmarinic acid	7.77 ± 0.23
8-OH salvigenin	3.81 ± 0.09
Chlorogenic acid	3.54 ± 0.51

## Data Availability

The original contributions presented in this study are included in the article. Further inquiries can be directed to the corresponding author.
